# The Persistence of Mode 1 Technology in the Korean Late Paleolithic

**DOI:** 10.1371/journal.pone.0064999

**Published:** 2013-05-28

**Authors:** Hyeong Woo Lee

**Affiliations:** Department Archaeology and Cultural Anthropology, Chonbuk National University, Jeonju City, South Korea; University of Florence, Italy

## Abstract

Ssangjungri (SJ), an open-air site with several Paleolithic horizons, was recently discovered in South Korea. Most of the identified artifacts are simple core and flake tools that indicate an expedient knapping strategy. Bifacially worked core tools, which might be considered non-classic bifaces, also have been found. The prolific horizons at the site were dated by accelerator mass spectrometry (AMS) to about 30 kya. Another newly discovered Paleolithic open-air site, Jeungsan (JS), shows a homogeneous lithic pattern during this period. The dominated artifact types and usage of raw materials are similar in character to those from SJ, although JS yielded a larger number of simple core and flake tools with non-classic bifaces. Chronometric analysis by AMS and optically stimulated luminescence (OSL) indicate that the prime stratigraphic levels at JS also date to approximately 30 kya, and the numerous conjoining pieces indicate that the layers were not seriously affected by post-depositional processes. Thus, it can be confirmed that simple core and flake tools were produced at temporally and culturally independent sites until after 30 kya, supporting the hypothesis of a wide and persistent use of simple technology into the Late Pleistocene.

## Introduction

**Figure 1:**
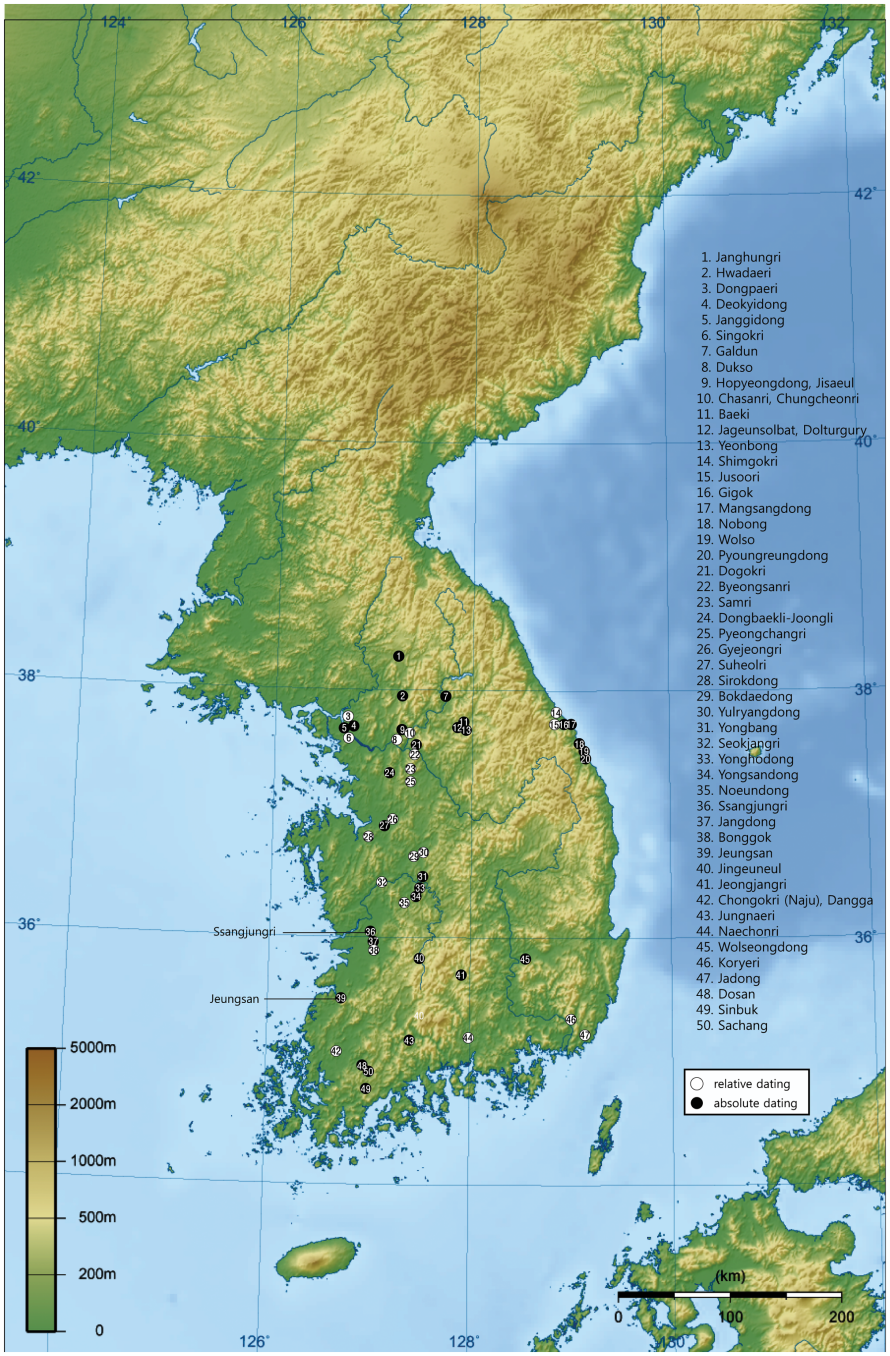
Locations of South Korean Paleolithic sites mentioned in the text. Note: The sites had been plotted on Korean peninsula topographic map [14].

**Figure 2:**
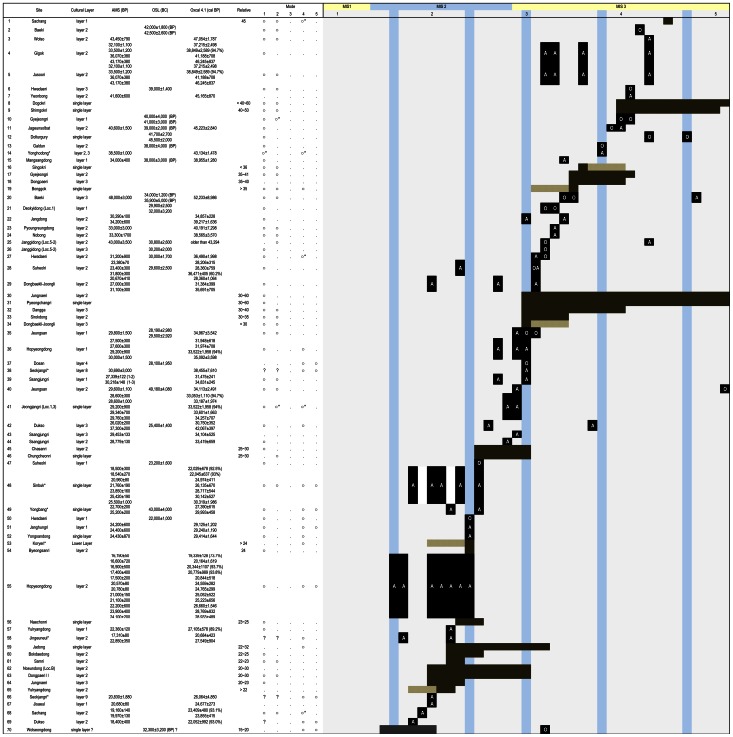
Selected Paleolithic sites in Korea. Representation of selected excavated Paleolithic sites yielding absolute and relative dates (from about less than 45 kya to more than 20 kya) in Korea. 1: [47], 2: [76], [77], 3: [78], 4: [79], 5: [80], 6: [8], 7: [77], 8: [81], 9: [82], 10: [83], 11: [84], 12: [76], 13: [77], 14: [85], 15: [77], 16: [77], 17: [83], 18: [86], 19: [87], 20: [77], 21: [88], 22: [20], 23: [77], 24: [89], 25: [90], 26: [90], 27: [8], 28: [73], 29: [72], 30: [48], 31: [91], 32: [48], 33: [92], 34: [1], 35: [44], 36: [7], 37: [48], 38: [2], 39: [15], 40: [44], 41: [6], 42: [93], [94] 43: [15], 44: [15], 45: [95], 46: [95], 47: [73], 48: [48], 49: [96], 50: [8], 51: [97], 52: [98], 53: [99], 54: [1], 55: [7], 56: [100], 57: [101], 58: [48], 59: [20], 60: [102], 61: [74], 62: [103], 63: [86], 64: [48], 65: [101], 66: [2], 67: [104], 68: [47], 69: [93], [94], 70: [105]. Note: The AMS dates quoted from primary references which are non-calibrated dates, while calibrating AMS dates obtained by OxCal 4.1.7 based on IntCal09 curve [22]. If not specified, the data are calculated at 95.4% probability levels. If not specified, AMS dates are addressed as B.P., while OSL dates are written as B.C. “*” on the mode sections is controversial for defining the technological mode due to typological classification, quantity or quality of data. On the graph, the figures inside the brackets represent calibrated AMS (A) and OSL (O). The sections horizontally shaded represent chronological data obtained by relative dating, where darker means more likely. The sections vertically shaded represent circa period of time for the Heinrich Events [106]. The Clark’s technological modes [17] are applied to determine the range of lithic technological variability [10] not for emphasizing distinctive and uppermost type fossils under the linear evolutionary scheme, thus the indicated modes can be plural in some cases.

There is growing evidence of simple core and flake tools in the Late Paleolithic of South Korea (see Figure 1 and 2). Under technological evolutionary schemes, these simple tools were regarded as the predecessors of blade-based assemblages. The initial period of blade-based assemblages in Korea is normally estimated to begin at 40 kya [1] or after 35 kya [2], and blades are replaced by microblade tools at about 25 kya [1], [2].

The period between 35–25 kya (the transitional period of MIS 3–2) is traditionally thought of as the Upper Paleolithic blade period [2]. Recently, this period was defined as the early part of the Korean Late Paleolithic instead of as the early part of the Upper Paleolithic due the absence of distinctive Middle Paleolithic tool types [3]–[5]. As a cultural marker, blades have been treated as the most significant tool type during the period up until the emergence of microblades; blade bearing sites have been mainly and selectively studied to explain the development of cultural traditions. The Korean sites of Jeongjangri (single layer) [6], Hopyeongdong (layer 1) [7], Hwadaeri (layer 1) [8], and Yongsandong (single layer) [9] are examples of blade-yielding sites, and simple tools found at these sites have generally been treated as insignificant or supplementary types. More complex forms of artifacts such as blades are considered to represent evolutionary progression, while more simple forms are regarded as “retentions of archaic/primitive components” following the Clark's technological modes [10].

However, recent studies have shown that simple assemblages comprising crudely made core and flake tools produced by direct percussion persisted into the Late Paleolithic in East Asia [2], [3], [11]. In several cases, such simple tools are not only incorporated in blade assemblages, but also appear independently during the coeval period with blade industries, even after 35 kya.

The survival of such simple tool assemblages over extensive periods of time offers the opportunity for new dimensional studies of modern human behavior during the transitional period. European and African technological sequence was not universal at all [12] and the blades technology is not inevitable single cultural maker to explain the Late Paleolithic assemblage in East Asia [13]. On the other hand, the simple tools have been viewed as a quantitative property of modern human behavioral variability [10]. This leads to the hypothesis that simple tools should be treated as important hallmarks within Korea and other parts of East Asia.

Despite such views, the sites that show independently occurring simple tool assemblages during the MIS 3–2 period in Korea have not received much attention. Ssangjungri (SJ) and Jeungsan (JS) are two recently excavated South Korean sites at which the artifacts are believed to have been discovered *in situ*. Both sites have generated accelerator mass spectrometry (AMS) dates, with optically stimulated luminescence (OSL) dating also performed at JS. Most of the artifacts at these two sites were recovered from stratified layers; the assemblages from the cultural horizons include choppers, polyhedrons, hammers, scrapers, and non-standardized flake tools. Technologically, all lithic tools show clear evidence of simple reduction sequences and typological variation, which do not differ from earlier assemblages. Therefore, secure chronological associations of lithic materials from SJ and JS surely contribute to the explanation of the extensive usage of simple lithic technology in temporal context.

## Materials and Methods

### 1-1. SJ, Background

In 2009, archaeologists working for the Jeolla Research Institute of Cultural Heritage discovered the SJ site at the edge of a hilltop in Iksan, South Korea [15]. The site was excavated in a series of trenches overlaid on a 40×20 meter square grid, and more than 600 stone artifacts have been excavated from the deeply stratified deposits. Most artifacts were recovered from stratified layers. Nine stratigraphic layers, including three cultural layers, were identified at the site, with a total thickness of about 6 meters. A total of 618 stone artifacts were excavated, with only a few being surface finds. Although three different cultural layers were found, it is not necessarily the case that there are three separate sets of lithic assemblages. Most of the lithics show cultural affinities with conventional Mode 1 lithic production, with some possibly Mode 2 types such as crude bifacially worked core tools. The technology applied is simple direct percussion, with artifact types such as choppers, polyhedrons, discoids and various forms of simply made flake tools.

SJ is located on a tributary of the Mankyung River. Around the Mankyung River basin (MRB), large numbers of Paleolithic sites have been recently discovered [16]. However, there are few systematically researched localities, and most MRB artifacts are surface finds. Around 60 Paleolithic find spots are known in the MRB, with most being from derived contexts.

The artifacts discovered in the MRB are divided into three scales of variation according to typological and technological change: simple core tools made of quartz and quartzite; blades; and micro-blade cores made of fine grained siliceous stones. In terms of Clark’s technological Modes [17], these assemblages might correspond to technological Modes 1, 4, and 5. Since these tools are from derived contexts, a detailed chronological pattern may be discerned from sites such as Sageunri [18], Bonggkok [19], and Jangdong [20]. These sites have generated absolute dates by AMS on soil. The separate cultural patterns have been observed; one is characteristic of Mode 1, with simple core tools; the other is Mode 4, with tools such as blades and tanged points. In addition, Mode 5 tools such as microblades have occasionally been discovered. Mode 4 assemblages are found in upper layers ranging from approximately 30 to 20 kya. However, most Mode 1 associated assemblages are not significantly older than Mode 4 and 5 assemblages.

None of the excavated MRB sites are high resolution. Bonggok is a single layer site that yielded about 400 artifacts, but mostly debitage, and few complete tools [21]. The site is loosely dated to 30 kya. The cultural horizons from Sageunri are mixed with recent deposits [18] and AMS results are also inconsistent. Such stratigraphic uncertainty is also seen at Jangdong, which has multiple cultural horizons similar to Sageunri. Some artifacts from Jangdong are believed to be associated with extensive colluvial activity, and suffered from post-depositional process [21]. Unlike these sites, SJ is a relatively high resolution site as comparatively large numbers of *in situ* artifacts with secure dates have been obtained. Therefore, evidence from the site of SJ is more substantial than that from other MRB sites.

### 1-2. SJ, Chronology

**Figure 3:**
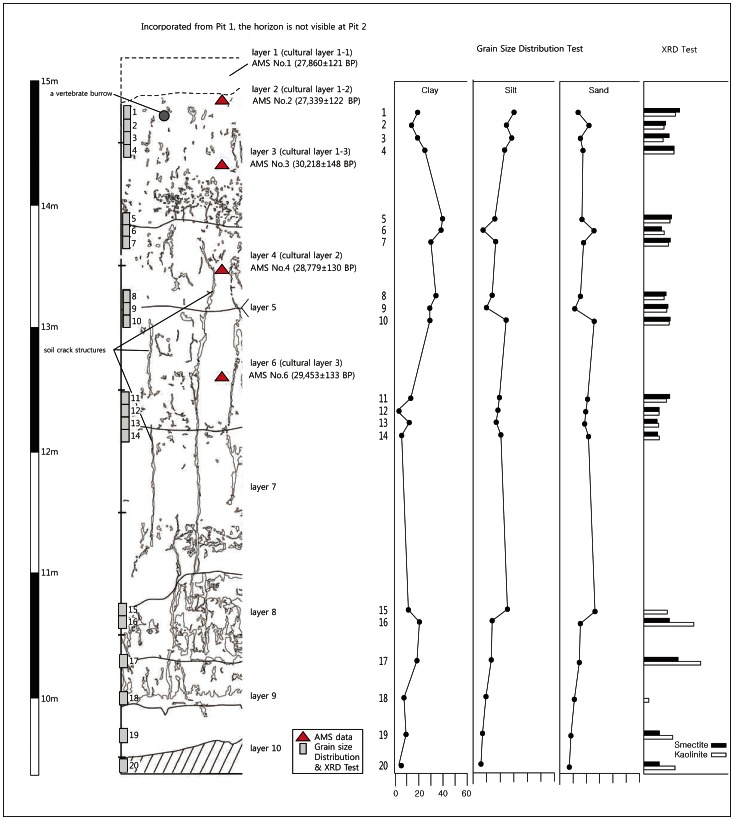
Schematic stratigraphic sequence based on Trench 2 at SJ. Note: One additional lithic bearing layer (cultural layer 1–1) was observed in Trench 1. The dates of cultural layer 3 are not consistent with the descending layers, although most of the AMS results indicate that deeper deposits are older. A possible reason why cultural layer 3 seems to be older than the layer below it is the mechanical reworking of organic matter and subsequent contamination. For further discussion, see [26], [36]. Courtesy of Jeolla Research Institute of Cultural Heritage, Korea.

SJ offers relatively rich Paleolithic evidence compared to previously researched sites in MRB. The numbers of artifacts are substantially larger, and no other site in the region has yielded a comparable number of artifacts. The stratigraphic layers are divided into 10 units, except for the disturbed top layer and artifact bearing horizons, which are divided into three. Cultural layer 1, which is the uppermost cultural layer, is subdivided into three sub-layers, 1–1, 1–2, and 1–3. The most prolific archaeological evidence can be seen in Trench (Pit) 2 (Figure 3).

In general, the cultural layers are more than 2 meters deep and are predominantly silt and clay. In addition, there are no clear signs of interrupted bedding structure within the units, such as aggradation of gravel or sandy material, so distinct stratigraphic breaks cannot easily be recognized. Below cultural layer 3, marked alternations to rocks or gravels are not visible, but sandy components increase with depth until reaching the weathered bedrock.

AMS determinations were carried out. These results were calibrated using OxCal v.4.1.7 based on IntCal09 database [22]. Cultural layer 1–2 dates to 27,339±122 B.P. (31,475±241 cal B.P.), while cultural layer 3 dates to 29,453±133 cal B.P. (34,104±525 cal B.P.). The thickness of the deposits from cultural layer 1–2 to 3 is approximately 2.5 to 3 meters, indicating that these layers formed during a relatively short period of time.

The general decreases in smectite and increases in kaolinite are related mainly to transformations to kaolinite under moist conditions. The low percentages or absence of smectite in the lower horizons indicates humid conditions. On the other hand, the increase in smectite in the upper horizons implies cold, arid conditions [15]. AMS data is only available for the upper layers, where the artifacts were recovered. These data provide an overall estimation of the transition from MIS 3 to MIS 2; thus, most deposits with artifacts seem to have been formed during not only cold but also arid conditions.

Additional supporting evidence for the association of cold episodes with artifact assemblages comes from the presence of soil wedges (soil crack structures that may be caused by repeated freeze-thaw activity) throughout the cultural layers. The interpretation of soil wedges is under ongoing debate in Korea [23]–[28]. These structures are often observed in Korean Paleolithic sites [29]–[31]. Moreover, polygonal structures are also commonly observed; in lateral view, these features link up to the form of soil wedge. In many Paleolithic sites, multiple sets of wedge structures are observed at different depths, penetrating several horizons.

When considering the width of the soil wedges from SJ it is too narrow for an ice-formed wedge as their width ranges from only 5 cm to 15 cm. These extraordinarily thin structures are easily interpreted as thin fissures resulting from desiccation [32]. Nevertheless, many Korean authors claimed that these structures formed under periglacial conditions during OIS 2 and 4 [25] or under cold and arid conditions [23], [26]. Normally, the younger wedges are attributed to MIS 2, while the older ones are attributed to MIS 4 [33]. With incorporated archaeological data, Chang claimed these younger wedges were formed from 25 to 22 kya [27].

However, it is questionable whether a true permafrost conditions existed in Korea during the Late Pleistocene. Furthermore, Bae claimed the structures from Korean sites are far from the conventional ice wedge structures formed under typical periglacial condition [34]. Lim and colleagues noted that periglacial conditions, which are the ideal conditions to form these wedge structures, were not prevalent in Korean peninsula. However, they argued that some soil wedges could be formed in times of “temporary” permafrost occurring during the last glacial maximum (LGM) [26]. Yu et al. offered an alternate explanation that the wedges (the more recent cracks) formed during either the cold and dry period of the last stage of MIS 3 or in the early stages of MIS 2 on the basis of another Korean Late Pleistocene site, Dukso [23]. A paleosol deposit, L1SS1, contained Aira-Tn (AT) tephra dated to about 25 kya [35] as well as a soil wedge in the upper portion of the L1SS1 deposit. Based on the evidence of that, cold and arid conditions were present during the artifact deposition period at SJ and the date seems to fall within early MIS 2 or slightly earlier.

### 1-3. SJ, lithic assemblage

**Figure 4:**
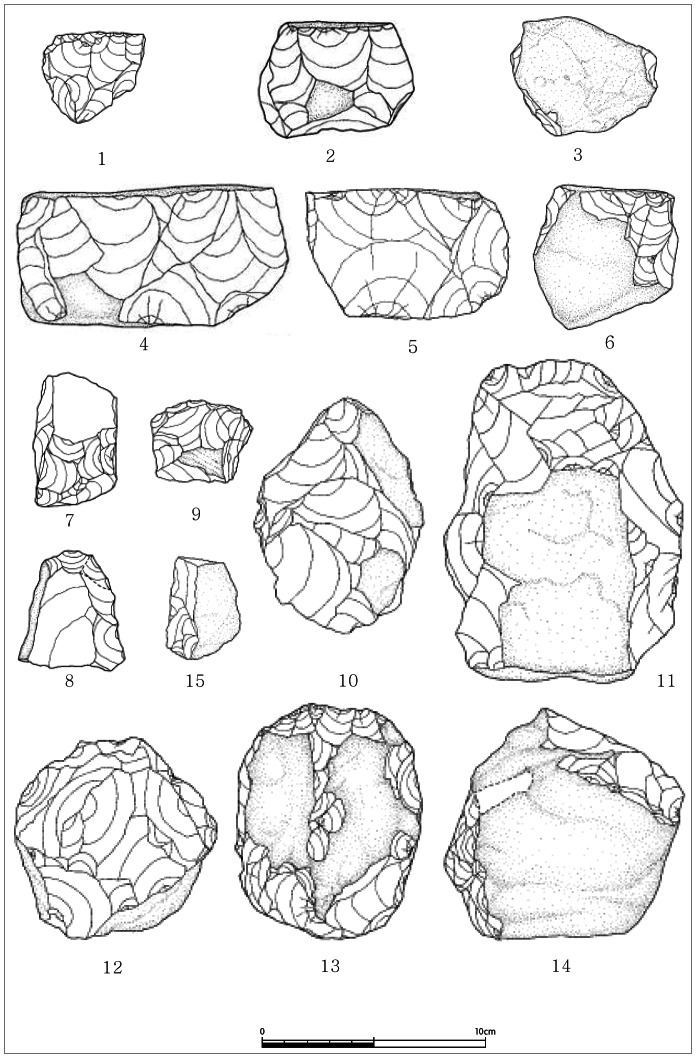
Tool types from SJ. 1. core, 2. single platform core, 3. scraper, 4. multi platform core, 5. core, 6–9. scrapers, 10–11. non-classic bifaces, 12. chopper, 13. non-classic biface, 14. plane, 15. scraper. Courtesy of Jeolla Research Institute of Cultural Heritage, Korea.

The frequencies of each tool type are described in Table 1. The assemblage is mainly composed simple cores and core tools primarily made from locally available quartzite (Figure 4). Despite the fact that nearly all of the artifacts were made from coarse-grained quartzite, which is believed to have been collected from the immediate surroundings of the site, the quality varies slightly. However, it is difficult to determine whether the tool makers deliberately altered the levels of refinement depending on raw material quality.

**Table 1:** Lithic types in the SJ assemblage.

layer/type	cores	flakes	debitage	non-classic bifaces	planes	large cutting tools	choppers	polyhedrons	core tools	side scrapers	end scrapers	denticulates	notches	awls	becs	hammerstones	anvils	blanks	total
surface collection	3	-	-	1	-	-	1	-	-		1	-	-	-	-	-	-	-	6
top layer (disturbed)	36	27	12	1	-	-		-	-	3	1	-	-	-	-	1	-	7	88
cultural layer 1-1	5	6	1	-	-	-	-	-	-		-	-	-	-	-		-	1	13
cultural layer 1-2	36	35	6	-	2	-	2	1	2	2	2	1	-	2	-	2	-	4	97
cultural layer 1-3	76	128	15	4	5	3	8	5	4	6	5	-	1	2	1	3	-	10	276
cultural layer 2	34	23	8	-	1	1	-	-	-	3	-	-	1	-	-	-	1	6	78
cultural layer 3	30	7	4	-	-	-	-	-	-	2	-	-	-	-	-	-	-	10	53
total	220	226	46	6	8	4	11	6	6	16	9	1	2	4	1	6	1	38	611

Note: A typological classification defined here is followed by the original excavators. The term of debitage used is morphological and technologically unidentified waste materials.

The basic flaking technique used was simple direct percussion. There is evidence of hammers and anvils, indicating that the artifacts were made on the spot and furthermore, that some of them must have been made using the finished block-on-block technique. Due to the use of relatively poor quality material, the impact scars on the surfaces are not clearly visible.

Formed artifacts are limited in number and characterized by a predominantly of an expedient knapping strategy. It is difficult to assess whether the tool makers tried to achieve a certain degree of standardization. Although simple core tools, such as choppers and other simplified core tools, predominate, they should not be classified under the conventional definition of a “pebble tool tradition” [37], [38] because of the apparent preference for large angular boulders rather than pebbles and cobble blanks, which are rounded clasts. The core tools are made on larger boulders, so a considerable amount of flaking is required. The cortex remaining on the finished tools is minimal, unlike conventional pebble core tools made of rounded clasts.

There are a relatively large number of exploited cores with removal scars. Faceted cores with little of the original surfaces are common. These seem to be the knapping residues considered as the end result of the usage of prepared striking platforms. Some SJ cores can be described as single, multi and radial platform cores (see Table 2), with the multi-platform cores dominant in the assemblage. Some of the single platform cores resemble so-called “horse-hoof” cores, which are found in Southeast Asia and Australia [39]. It is premature to argue whether these are similar to single platform blade cores [40] or conventional early stage cores [41]. Single, multi, and radial cores represent various points in the flake removal sequence, and therefore the simplicity of the finished tools does not automatically imply simplicity of knapping techniques [42]. Nonetheless the lithic technology at SJ falls into Mode 1 or Mode 2 *sensu lato* because of presence of crudely made bifacially worked core tools.

**Table 2:** Three different core types from SJ.

layer/type	single platform cores	multi platform cores	radial cores
surface collection	-	-	-
top layer (disturbed)	8	29	-
cultural layer 1-1	2	3	-
cultural layer 1-2	10	30	3
cultural layer 1-3	13	80	12
cultural layer 2	8	26	2
cultural layer 3	6	24	1
total	47	192	18

The presence of Modes 1 and 2, or a pebble tool assemblage and a (non-classic) bifacial handaxe assemblage, are not necessarily mutually exclusive [43] in a Korean context. Many sites indicate that these two assemblages occurred contemporaneously in Korea. Technological stagnation is suggested by the fact that the bifacially worked core tools are crude pseudo-handaxes or handaxe-like core tools. They are not intensively worked, are not perfectly symmetrical, and are very thick in cross-section. In total, six bifacially-worked core tools were discovered at SJ; four of them in cultural layer 1–3, one in a disturbed by agricultural practices, and one on the surface.

### 2-1. JS, Background

JS is another open-air site located about 70 km from SJ. To the east, there are mountains and to the west, the terrain is flat [44]. JS is located about 20 km away from a major river system, the Yeongsan River. Systematic Paleolithic excavations and full scale field surveys have not yet been conducted around the site, so there are few data from the Paleolithic site. However, a few high resolution sites have been reported on the Yeongsan River, such as Dangga [45], Dosan [46], and Sachang [47] which range in age from MIS 3 to MIS 2. The artifacts found at these sites include choppers, spheroids, handaxes and various forms of flake tools [48].

The Honam Cultural Property Research Center carried out an excavation. In total, seven localities and about 7,300 square meters were excavated. Over 1,800 lithic artifacts were discovered in a stratified context at JS. The chronology and assemblage patterns are similar to those at SJ. Artifacts from multiple layers show rather homogeneous Mode 1 assemblage patterns alongside non-classic handaxes.

### 2-2. JS, Chronology

**Figure 5:**
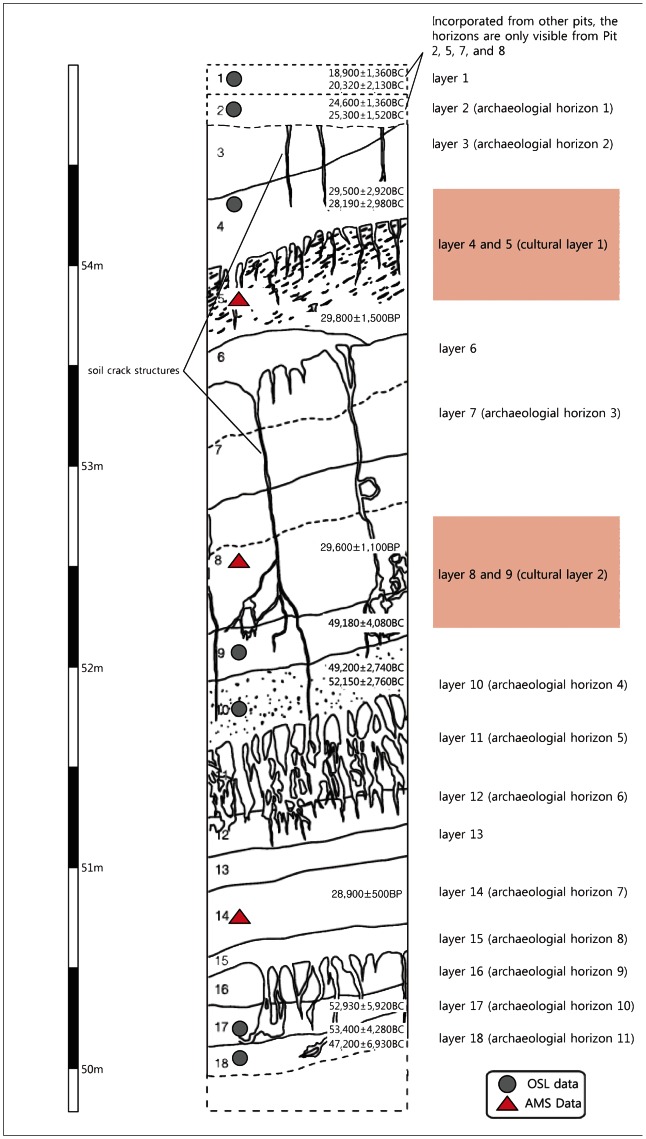
Schematic stratigraphic sequence based on Trench 1 at JS. Courtesy of Honam Cultural Property Research Center, Korea.

The site of JS has about 6 meters of stratified deposits and is comprised of 19 stratigraphic units including the uppermost layer (Figure 5). The horizons were not classified through systematic studies of sediments and soil stratification or bedding structures. Instead, the units were divided by visual and texturally homogeneous or gradational conditions. In consequence, it is difficult to reconstruct the depositional environment. However, absolute dating has been conducted and may be used to reconstruct the primary chronological sequence.

Due to the lack of grain size analysis, it is difficult to precisely describe the geomorphologic features of the site. However, all of the artifact bearing horizons are predominated by silt and clay, and no marked stratigraphical breaks are found at the site. Clear stratigraphic markers of intervals cannot be determined; further, brownish or reddish silt and clay features occur repeatedly. The artifact horizon profile is like an enlarged version of SJ; the artifact-bearing units of JS are about 6 meters deep, while those of SJ are about 2 meters deep.

The assemblage is characterized by mostly simple core and flake tools regardless of horizons. Except for the uppermost layer, all artifacts were highly likely to be made of locally obtained quartzite. These are choppers, polyhedrons, and simple flake tools such as scrapers. Among the horizons, many artifacts were refitted after excavation. Artifacts from archaeological horizons 4 and 5, and 8 and 9 conjoin with each other. Thus, a re-classification of the stratification at JS is necessary. Layers 4 and 5 were renamed cultural layer 1, and layers 8 and 9 were renamed as cultural layer 2. Other artifact-bearing layers are referred to as “archaeological horizons (AH)”.

Throughout all of the deposits, sand and silts dominate the sequences. Pollen analysis was performed, but the results were not significant. Only a small amount of pollen was obtained from the lower deposits, which comprise layers AH 8 to AH 5 and are located below the most prolific and significant artifact-bearing layers (cultural layers 1 and 2). Therefore, pollen analysis was not useful for understanding the environmental conditions or the stratigraphic succession of the deposits. Although pollen from pine, oak, and hazel were found, it was too sparse to provide a detailed environmental reconstruction. The reason for the paucity of pollen data might be due to the successive recurrent arid conditions and oxidization effects [44].

Absolute dating was conducted by AMS and OSL. The samples for AMS were collected from geological layer 5, while samples for OSL were obtained from geological layer 4. The AMS data indicate a date of 29,800±1,500 B.P. (34,967±3,542 cal B.P.), while OSL data indicate 28,190±2,980 B.C. and 29,500±2,920 B.C. These two series of chronological data suggest an estimated age of around 30 kya; this age range indicates that the cultural layer was deposited during the transitional period between MIS stages 3-2. However, the results of AMS and OSL dating for cultural layer 2 are seriously inconsistent. The sample for AMS was collected from geological layer 8, indicating a date of 29,600±1,100 B.P. (34,113±2,491 cal B.P.), while the sample for OSL was obtained from geological layer 9, and dated 49,180±4,080 B.C.

All samples were sediments and not bone or charcoal, which are normally preferred for accurate dating. However, the AMS results from the three stratigraphic units indicate a narrow time range, representing a range slightly older than 30 kya according to the calibrated AMS ages. On the other hand, another set of absolute dates (OSL) produced a very wide time range. The full-fledged OSL dating for archaeological researches was introduced only recently in Korea, and there has been growing interest in the absolute dating of Paleolithic sites. However error may occur when dating sites with poor exposure to daylight prior to deposition [49].

Multiple absolute AMS and OSL dates are known from a very limited number of sites. Although each layer from Hwadaeri offers consistent results from both dating techniques [50], inconsistency occurs even more frequently at other sites. Figure 2 illustrates some of the multiple dating results from Korean Paleolithic sites. Most results are inconsistent and at many sites, the time range suggested is too large to be acceptable. AMS dating in Korea is also considered problematic [29]. In spite of these problems, AMS is a better established absolute dating technique than OSL. Therefore, AMS dates are considered more acceptable, while OSL can be used as a supplementary type of chronological data.

Recently, Seong categorized existing radiocarbon dates in Korea [51], and reevaluated the data on the basis of qualitative and quantitative measurements which is the simplified version of the original criteria proposed by Pettitt et al. [52] and Graf [53]. First, he assessed sample type, number of correlated dates, and standard deviations. Then, he interpreted other valid geological archaeological data as “accepted”, “tentative”, and “rejected”. Under this scheme, the “accepted” radiocarbon dates make up less than one third of the total of 135 dates known from Korea [51]. According to these criteria, the radiometric dates from cultural layer 1 and 2 are not “accepted”, while the data from cultural layer 1 seems to be not much away from “tentative”.

It is more difficult to determine the credibility of the age of cultural layer 2. However, the artifact types and narrow span of AMS dates imply that layer 2 is broadly contemporary to cultural layer 1. Similarities in the soil textures and environmental conditions between SJ and JS also support their temporal homogeneity. Another (although not exact) convincing evidence lies in the soil wedges (crack structures) cutting through the artifact-bearing horizons at both sites. If these structures represent repeated freeze-thawing under cold and arid conditions, both sites may have been occupied under the same conditions. The archaeological horizons below cultural layer 2 are even more difficult to verify. Lithic materials are consistent with those found in upper horizons, and the AMS dates yield a similar time span, earlier than 30 kya. Again, OSL dates do not seem to use to support claims a narrow depositional time span at this site.

### 2-3. JS, lithic assemblage

The frequencies of each tool type found at JS are summarized in Table 3. Most artifacts are expedient tool types made of conventional raw materials such as quartzite, although they are recovered from various stratigraphic levels. The lithic evidence from JS is collectively described as an enlarged assemblage of SJ because it contains large quantities of almost identical types of tools. Therefore these two sets of finds can be interpreted as having temporal and technological integrity. More than 1800 artifacts were found, the number of shaped tools is not so large. The majority among shaped stone tools is simple core tools, such as choppers, polyhedrons, and simple flake tools, which lack standardized shapes. Most are made of locally available quartzite cobbles and boulders. The industry is characterized by crude, rough flake reduction reflected in situational improvisational lithic manufacture.

**Table 3:** Lithic types in the JS assemblage.

Layer/Type	cores	flakes	blade	debitag	non-classic bifaces	picks	large cutting tools	choppers	planes	polyhedrons	Misc. core tools	side scrapers	scraper pieces	end scrapers	denticulates	notches	knives	points	awls	becs	hammerstones (inc. battered cores)	anvils	total
top layer (disturbed)	2	7	-	-	-	-	-	1	1	1	-	-	-	-	-	2	-	-	-	-	-	-	14
AH 1	-	2	1	-	-	-	-	-	-	-	-	-	-	-	-	-	-	-	-	-	-	-	3
AH 2	-	3	-	2	-	-	-	-	-	-	-	-	-	-	-	2		-	-	-	-	-	7
cultural layer 1	74	833	-	253	3	1	2	15	6	2	3	30	2	9	9	54	4	-	9	3	19	2	1333
AH 3	-	5	-	5	-	-	-	1	-	-	-	-	-	-	-	-	-	-	-	-	-	-	11
cultural layer 2	5	63	-	27	1	1	1	4	3	1	2	7	-	2	1	3	1	1	1	-	3	-	127
AH 4	6	37	-	13	-	1	1	1	1	3	1	3	-	1	-	3	-	1		-	1	-	73
AH 5	10	44	-	11	-	-	-	4	5	5	2	4	-	-	3	2	-	-	1	-	4	-	95
AH 6	5	13	-	1	-	-	2	2	2	5	2	-	-	1	-	-	-	-	-	-	1	-	34
AH 7	2	6	-	2	-	-	-	-	-	-	1	-	-	-	-	1	-	-	-	-	-	-	12
AH 8	-	-	-	-	-	-	-	1	-	1	-	-	-	-	-	-	-	-	-	-	1	-	3
AH 9	11	37	-	13	1	-	1	1	3	3	-	1	-	1	-	-	-	-	2	-	4	-	78
AH 10	2	26	-	11	-	4	3	1	1	2	-	4	-	3	1	2	-	-	-	-	3	1	64
AH 11	-	-	-	-	-	-	-	-	-	1	-	-	-	-	-	-	-	-	-	-	-	-	1
AH 12	-	5	-	4	-	-	1	-	-	-	-	-	-	1	-	2	-	-	-	-	-	-	13
Total	117	1081	1	342	5	7	11	31	22	24	11	49	2	18	14	71	5	2	13	3	36	3	1868

Note: A typological classification defined here is followed by the original excavators. The term of debitage used is morphological and technologically unidentified waste materials. Some polyhedrons might be identified as sub-spheroids, present author try to keep the data recorded by the original excavators.

In contrast to the contextual homogeneity of artifacts type and raw material usage pattern, a single blade was found in the top layer (a disturbed unit), and was chronologically and culturally separate from the rest of the artifacts. Along with two other flakes that were associated with the blade, they were made of fine grained siliceous shale. In Korea, siliceous shale (often referred to as rhyolite by Korean researchers) artifacts are commonly found within Late Paleolithic contexts [1]. These types of raw materials were probably not available near JS, and therefore the acquisition of these raw materials was probably not local. Except for these three artifacts, no further exotic artifacts were found in the deposits.

**Figure 6:**
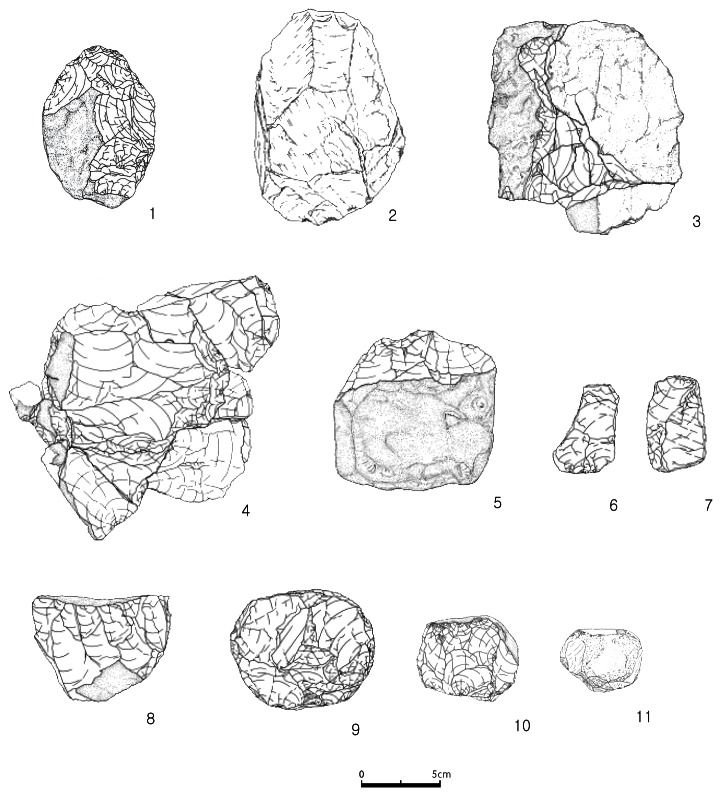
Tool types from JS. 1–2. non-classic bifaces, 3–4. conjoining pieces, 5. chopper, 6–7. end scrapers, 8. single platform core, 9–10. polyhedrons, 11. hammerstone. Courtesy of Honam Cultural Property Research Center, Korea.

Thought to be typologically and technologically assignable to a Mode 1 assemblage with a simple reduction strategy, the incorporated handaxe shaped core tools offer further attention for the possibility of an extended handaxe (non-classic form) occupation up until the Late Pleistocene period (Figure 6).

Further investigation is required to determine whether these bifacially worked core tools are typical Acheulean handaxes. Only five bifaces of quartzite were discovered, although careful excavations had been carried out. The low percentage of bifaces at JS (same as those from SJ) is thought to be a reminder of a part of the general observations (the rarity of bifaces) of the eastern Movius Line [54], [55]. In terms of morphological features, all are bifacially flaked however, the lack of a refined symmetrical form and a thick cross section indicates hard-hammer percussion, and no strong evidence of being an authentic Acheulean handaxes. The handaxe cutting edges from JS and SJ do not extend around their circumferences, and moreover, the tips and butt are poorly prepared. Hence, it is very difficult to argue that these features show effective large and sharp cutting edges.

From the assemblage from JS and SJ, artifacts that may have been used for pounding, are common (see Table 3). Typologically diagnosed as some cores, choppers, and polyhedrons, even planes with battering signs are suggested as the pounding implements, although the ideal pounding pieces, which are spheroids [56], are not clearly observable. If collections of identified hammerstones are treated as a percussive gear for exploitation food resources as well as making tools, pounding-related tools are more in evidence than sharp cutting edged tools. Picks, handaxes, and other miscellaneous cores with large cutting edges are significantly fewer.

Refitting analyses have been carried out at the site. The highest numbers of refitted artifacts are concentrated in cultural layer 1. Of total number of sets (n = 113), 98 sets were recovered from cultural layer 1 (Table 4). They show the spatial concentrations of refitted pieces, suggesting a primary site with minimal post-depositional process. In order to reconstruct the sequence of knapping, conjoining fragments were classified as core, flake, debitage etc. Although full reconstructions are not yet possible, refitting analyses provide information about the basic knapping strategies used at the site. Conjoining sets comprised of flakes and debitage are abundant; however, cores and core tools are also incorporated in refitting elements. The presence of cores with various forms of flakes and residues suggest that all the major tool reductions were carried out at the site. More synthetic studies are required to clarify whether fragments represent breakage by post- depositional processes or the consequences of reduction [57]. Fragments seem to have been produced with minor effort and minimal technological expertise.

**Table 4:** Summary of individual refitting pieces from cultural layer 1, JS.

set	c	f	d	ft	ct	o	set	c	f	d	ft	ct	o	set	c	f	d	ft	ct	o
16	1	10	-	-	-	-	49	-	1	-	1	-	-	82	-	1	-	1	-	-
17	1	5	-	-	-	-	50	-	-	5	-	-	-	83	-	12	-	1	1	-
18	1	1	3	-	1	-	51	-	-	4	-	-	-	84	-	5	-	1	1	-
19	1	6	-	2	-	-	52	-	-	4	-	-	-	85	-	2	-	-	1	-
20	1	6	-	1	-	-	53	-	4	-	-	-	-	86	-	2	-	-	-	-
21	1	4	-	-	-	-	54	-	2	-	-	-	1	87	-	8	-	1	-	-
22	1	3	-	-	-	-	55	-	1	-	2	-	-	88	-	1	-	1	-	-
23	-	-	3	-	-	-	56	-	3	-	-	-	-	89	-	4	-	3	-	-
24	1	2	-	-	-	-	57	-	2	1	-	-	-	90	-	1	-	1	-	-
25	1	1	-	-	-	-	58	-	2	-	-	-	-	91	-	1	-	1	-	-
26	1	1	-	-	-	-	59	-	2	-	-	-	-	92	-	1	-	1	-	-
27	1	1	-	-	-	-	60	-	2	-	-	-	-	93	1	8	-	1	-	-
28	1	1	-	-	-	-	61	-		-	1	-	1	94	-	1	-	1	-	-
29	-	-	-	2	-	-	62	-	-	4	-	-	-	95	-	11	-	1	-	-
30	-	2	-	1	-	-	63	1	1	-	2	-	-	96	-	1	-	1	-	-
31	1	13	-	-	-	-	64		3	-	-	1	-	97	-	5	-	-	-	-
32	-	-	6	-	-	-	65	-	1	-	-	1	-	98	-	4	-	-	-	-
33	-	-	5	-	-	-	66	-	1	-	1	-	-	99	-	3	-	-	-	-
34	1	2	-	-	-	-	67	-	2	-	2	-	-	100	-		3	-	-	-
35	-	1	-	-	1	-	68	-	3	2	-	-	-	101	-	3	-	-	-	-
36	-	4	-	1	1	-	69	-	1	2	-	-	-	102	-	3	-	-	-	-
37	-	1	-	1	1	-	70	-	3	-	-	-	-	103	-	2	-	-	-	-
38	1	3	-	1	-	-	71	-	2	-	-	-	-	104	-		2	-	-	-
39	-	-	-	2	-	-	72	-	2	-	-	-	-	105	-	2	-	-	-	-
40	1	-	-	3	-	-	73	-	2	-	-	-	-	106	-	2	-	-	-	-
41	-	1	-	2	-	-	74	-	1	1	-	-	-	107	-	2	-	-	-	-
42	-	6	-	2	-	-	75	1		-	1	-	-	108	-	2	-	-	-	-
43	-	5	-	1	-	-	76	-	2	-	-	-	1	109	-	2	-	-	-	-
44	-	3	-	1	-	-	77	1	1	-	-	-	-	110	-		1	-	-	1
45	-	6	-	2	-	-	78		2	2	1	-	-	111	-	1	-	-	-	1
46	-	1	-	1	-	-	79	2	8	-	2	-	-	112	-	-	-	2	-	-
47	-	3	-	1	-	-	80	1	6	-	-	-	-	113	-	3	-	-	-	-
48	-	1	-	1	-	-	81	1	1	-	1	-	-							

Note: c (core), f (flake), d (debitage), ct (core tool), ft (flake tool), o (others). The numbers on the first, eighth and fifteenth columns are serial number. The classification of types of forms is followed by the original excavators. Conjoined fragments demonstrating as the results of events of considering natural breakages were omitted. The pieces more or less likely to break in use are included (e.g. hammerstones).

The proportion of the cortical and non-cortical pieces partly illustrates a lithic exploitation pattern [58]. From evidence of the refitted pieces, the non-intensive lithic exploitation can be addressed from the evidence of a larger proportion of cortical pieces and small series of flakes removed from the cores. One of the exceptional cases is a set including a handaxe (No. 83 on Table 4). The set comprises 12 flakes, one flake tool, and one handaxe. These pieces do not represent a complete reduction sequence because the cortical parts are limited. Related flakes show mainly rough outs rather than thinning flakes that might suggest refined modifications or further re-sharpening. The largest set is No. 31, which shows 13 pieces of flake and 1 core. Nonetheless, the number of refitted pieces per set is far fewer in general (see Table 4). From this evidence, handaxes might have been made with a relatively more attention than any other implements. Beside the information of handaxe related pieces, most sets could have been made under less considerable time and energy; again, an expedient reduction strategy was dominant at JS.

## Results and Discussion

Based on the Paleolithic evidence from SJ and JS, these assemblages can be described as follows.

1. highly expedient lithic reduction strategy

2. on-site use of poor quality raw material regardless of blank form

3. relatively high frequency of pounding forms

4. presence of bifacial tool forms

5. lack of diagnostic criteria associated with LCT or small tool traditions

First, the suggested age of the SJ and JS sites is about 30 kya, when blades and associated toolkits had been already introduced to the Korean peninsula. Nevertheless, the simple technical systems observed at these sites indicate that non-predetermined tool forms prevailed. These artifacts are not strongly diagnostic tool types and are not representative of any given temporal unit. They are minimally modified with little secondary retouch, and the working edges are irregular and overall poorly made. No objective data supporting refinement of shape are available, and thus it is impossible to support the hypothesis of progressive lithic complexity over time.

Second, the pattern of acquisition of raw material at these sites indicates minimal preparation. Most of the tools were made of locally available coarse grained rocks. Since the implements were made in a variety of expedient ways, selective pressure for intense resource exploitation does not seem to have been present. The shapes of the raw materials also do not strongly correspond to specific artifact types. The patterns of use do not conform to "pebble tool tradition" in the strict sense [38], because strong or pervasive preferences for pebble forms are not apparent.

Third, implements suggested that pounding technology is more clearly observed. Although the perfect spheroids to be responsible to the ideal percussor are not examined at the sites, the relative gravity of the tools for pounding as opposed to the tools for cutting never diminished. Originally classified as some polyhedrons (it should bear in mind that the classification of lithic types in this paper is followed by the original excavators’ decision) can be treated as the sub-spheroids or faceted spheroids, because their multiple angles and ridges are not very clear. They also show non-preservation of cortical surfaces with developing round shapes through battering.

At the two sites, large amounts of seemingly pounding related types of implements have been found. The hammerstones at SJ (n = 6) and the hammerstones and battered cores at JS (n = 36) (Table 1 and 3) are not insignificant proportions in comparison with the actual figure of formed artifacts within the sites. These seem to have served as substitutes for pounding activities. Normally, polyhedrons and sub-spheroids are regarded as exhausted cores from flake manufacture [59], [60]; hence, it cannot be directly linked to the pounding activities. Nonetheless, they cannot be counted as cutting tools [61]. As a result, tools for cutting, not pounding, are becoming more marginal.

One thing to consider is that some typologically classified choppers, polyhedrons, and cores from SJ and JS show marked signs of bruising/battering the tool surfaces. Since most tools are not rolled, the primary bruising/battering agent could not have been post-depositional. This evidence suggests that pounding activities were conducted routinely at these sites.

**Figure 7:**
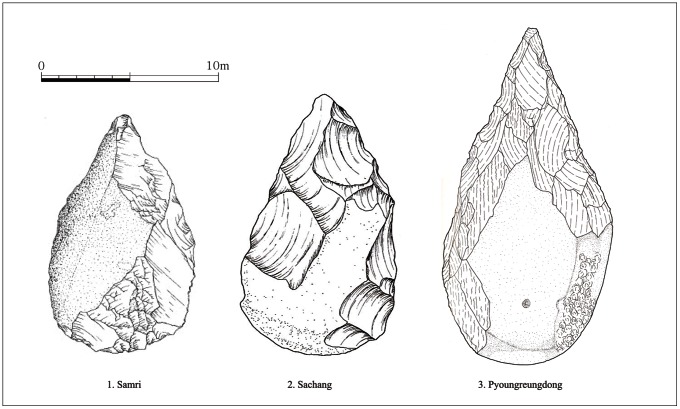
Examples of the Late Pleistocene bifacially worked core tools in Korea. 1. Samri [74], 2. Sachang [47], 3. Pyoungreungdong [75].

Fourth, as parts of simple tool assemblages, the persistence of bifaces is also considered. The poor manufacture of the bifaces from SJ and JS have produced strong doubt about their authenticity. However, the presence of some rather refined bifaces from not the Middle but the Late Pleistocene sites (Figure 7) seems to indicate a new directional thought. Although complete metric data for Late Pleistocene Korean bifaces are not yet available, many of them from various sites are expediently made and are attributed to atypical or non-classic types. At the same time, more or less symmetrically made refined forms are also found from roughly coeval sites along with SJ and JS. The bifaces from Korea have recently been reevaluated, and their interpretations have focused not on their presence and absence [62] but on qualitative and quantitative comparisons with African and Eurasian Acheulean handaxes [54], [55], [63], [64]. Further, it is normally claimed that Korean bifaces do not always share the characteristic features of bifaces from the west of the Movius Line. Nevertheless the existence of artifacts with affinity to Mode 1 alongside handaxes is an interesting feature which needs to be further explored.

Last, the distinction between large cutting tool and small tool traditions should be reexamined. Seong argued that the handaxe-chopper-polyhedral dominant tradition (HCPT) persisted until MIS 3, while the small quartzite artifact dominant tradition (SQAT), without bifaces, started in MIS 3 [11]. Such tool size distributions seem to depend on the availability of suitable raw material size and shape [65], so this might not be relevant with the distinctive organization of lithic technology. Since the data from SJ and JS do not show significantly small or large tools, they cannot be assigned to these two traditions. Indeed, if a certain preference for size or pattern is observed, it may be due to situational factors that may re-occur under similar conditions [66].

For the last several decades, the general concept of East Asian lithic variation has traditionally been summarized as a handaxe tradition in the west and a chopper-chopping tool tradition in the east [67], [68]. In addition to the overall geographical framework, the nature of temporal changes in technological and typological variation has been debated recently. The question of the Middle Paleolithic validity in East Asia, including Korea [3], [5], is addressed in the context of the preferred twofold cultural period model, which includes data from the Early Paleolithic (simple forms such as choppers and bifaces) and that of the Late Paleolithic (refined blade and microblade forms) due to the absence of any distinctive Mode 3 technocomplex elements [1], [5].

The conventionally synthesized western cultural chronological model has been challenged by regionally specific geographical and temporal frameworks. The scale of lithic variation comprised by the conventional technological modes does not support the hypothesis of continuous cultural evolution [10], [69]. Instead of a continuum of variation in a unilinear evolutionary model, recursive behavior changes [10], [70], [71] under recurrent contextual situations [66] better explain the unique differences from western patterns observed in eastern assemblages.

The accumulated Paleolithic data for Korea is mature enough to provide this alternative perspective against from the traditional model of cultural transition. The last 10 years of data for the Korean Late Paleolithic largely indicate that two separate technocomplexes (simple reduction sequences for producing crude core tools and more complex reduction sequences for producing blades and microblades) are equally important, because the parallel existence of the two technocomplexes is a better explanation than the succession of one after the other [11]. The appearance of blade technology seems to take place the end of MIS 3. However, earlier simple toolkits were not completely replaced newer tool forms, but persisted after the introduction of blade assemblages [3]. Because blades are considered to be significant cultural and chronological markers, blade-bearing sites are naturally highlighted. However, attention must be turned to the other end in order to clarify late human behavior in Korea.

Hwadaeri and Hopyeongdong are known as the sites for representative of successive technocomplex shift from simple to complex lithic reduction [3]. Hwadaeri shows the three stratified cultural deposits were dated by AMS and OSL and show from non-blade to blade related toolkits. Cultural layer 3 (39 kya by OSL) yielded simple choppers, polyhedrons, and crudely made flake tools, whereas the upper layers at the site correspond to cultural layer 2 (about 31 kya by non-calibrated AMS and 30 kya by OSL) and cultural layer 1 (22 kya by OSL) yield blades and tanged points [8]. Hopyeongdong yields blades and microblades from a stratified series of Late Pleistocene deposits [7]. Extensive geological and chronological studies at this site revealed two cultural layers that show a technological shift from blades (the oldest date about 30 kya by non-calibrated AMS) to microblades (the oldest date about 24 kya by non-calibrated AMS) (Figure 2).

Taking the evidence from the two sites of Hwadaeri and Hopyeongdong, successive technocomplex changes from the simple to the refined, as well as more refined tool forms, are observed. However, a majority of the tools from both sites are not blades or microblades. The proportions of blades are consistently low throughout the Korean Late Paleolithic sites [1]. Blades never have formed a large percentage of the assemblage at any Korean site, and a majority of tool types are simple tools. Evidence of bladelet production is relatively sparse, and true prismatic blade technology is rarely observed during 30 kya.

In addition, simple core tool assemblages occur independently and continuously at many sites during the blade and even microblade periods. The sites of Jangdong (cultural layer 2, the date about 30 kya and 34 kya by non-calibrated AMS), Suheolri (cultural layer 2, circa 30 kya by OSL), and Dongbaekli-Joongli (cultural layer 2, the oldest one about 31 kya by non-calibrated AMS) yielded only simple Mode 1 affinity artifacts with no other higher complex tool forms observed [20], [72], [73] (Figure 2). Simple stone flaking should be equally highlighted during the early stages of the Late Paleolithic in Korea.

Most tools of SJ and JS can be explained as “instant technology” with minimal curation [56]. However, the simple and static nature of the lithic record may not be culturally meaningful under a predetermined trajectory, but can be regarded as a versatile technological strategy specific to environmental conditions with very recursive behavior [10]. The predominance of simple tools might indicate the spontaneous and improvised raw material exploitation pattern. And the variation in sizes of finished forms and the large number of non-cutting tools suggest highly situational lithic reduction strategies. More importantly, this lithic system was not unsuccessful. Paleolithic data around 30 kya indicate that this system was not saltationally replaced by blade-related tools that require considerable skill to fabricate.

## Conclusion

To summarize the lithic variation observed at SJ and JS during the early Late Paleolithic in Korea: blades are present, but the persistence of simply made core and flake toolkits is an integral part of the Late Paleolithic Korean assemblages. In a traditional view, the late occurrence of simple tool assemblages has been considered as a subordinate source of data in the shadow of the Mode 4 or 5 prismatic blade and microblade assemblages that have been treated as a cultural and chronological “type fossils”. There are growing numbers of simple core and flake tool assemblages known from Korea, which are comprised mainly of Mode 1, such as the technocomplex toolkits, with a minority of Mode 2 like assemblages. The chronological distributional pattern of the assemblages on Figure 2 consists of artifacts found in stratigraphic contexts and associated with relative and absolute dates. These data highlight the dominant feature of simple reduction strategies until the early Korean Late Paleolithic. The technological attributes from SJ and JS share the traits of many other Korean sites possessing Mode 1 and diminutive Mode 2 features. So far, data indicate that Mode 4 technocomplex was not a very strong technological breakthrough, across the Korean peninsula.

The persistence of simple lithic reduction sequences until the later periods in Asia, particularity Southeast and East Asia, indicates that Clark’s implications about different technological modes representing evolutionary progress must be challenged [10]. The prevailing conservatism without any clear signs of divergence between Mode 1 and 2 industries is an important parameter in this context as well, and the evidence from SJ and JS will contribute to an understanding of this debate.

However, several problems remain to be overcome. Efforts should be made to find more high resolution archaeological sites and more secure chronometric ages to improve the analysis proposed here. Although the persistence of simple toolkits in Korea is observed, this archaeological pattern has not been fully explained yet, but this must be addressed in the future.
